# Access to social capital and risk of HIV infection in Bukoba urban district, Kagera region, Tanzania

**DOI:** 10.1186/2049-3258-72-38

**Published:** 2014-11-03

**Authors:** Gasto Frumence, Maria Emmelin, Malin Eriksson, Gideon Kwesigabo, Japhet Killewo, Sabrina Moyo, Lennarth Nystrom

**Affiliations:** Department of Development Studies, Muhimbili University of Health and Allied Sciences, PO Box 65454, Dar es Salaam, Tanzania; Department of Clinical Sciences, Social Medicine and Global Health, Lund University, Malmö, SE-205 02 Sweden; Department of Public Health and Clinical Medicine, Epidemiology and Global Health, Umeå University, Umeå, SE-901 85 Sweden; Department of Biostatistics and Epidemiology, Muhimbili University of Health and Allied Sciences, PO Box 65015, Dar es Salaam, Tanzania; Department of Microbiology, Muhimbili University of Health and Allied Sciences, PO Box 65001, Dar es Salaam, Tanzania

**Keywords:** Structural, Cognitive social capital, HIV prevalence, Tanzania

## Abstract

**Background:**

Kagera is one of the 22 regions of Tanzania mainland, which has witnessed a decline in HIV prevalence during the past two decades; decreasing from 24% in 1987 to 4.7 in 2009 in the urban district of Bukoba. Access to social capital, both structural and cognitive, might have played a role in this development. The aim was to examine the association between individual structural and cognitive social capital and socio-economic characteristics and the likelihood of being HIV infected.

**Methods:**

We conducted a population-based cross-sectional study of 3586 participants, of which 3423 (95%) agreed to test for HIV following pre-test counseling. The HIV testing was performed using enzyme-linked immunosorbent assay (ELISA) antibody detection tests. Multiple logistic regression analysis was applied to estimate the impact of socio-economic factors, individual structural and cognitive social capital and HIV sero-status.

**Results:**

Individuals who had access to low levels of both structural and cognitive individual social capital were four and three times more likely to be HIV positive compared to individuals who had access to high levels. The associations remained statistically significant for both individual structural and cognitive social capital after adjusting for potential confounding factors such as age, sex, marital status, occupation, level of education and wealth index (OR =8.6, CI: 5.7-13.0 and OR =2.4, CI: 1.6-3.5 for individual structural and cognitive social capital respectively). For both women and men access to high levels of individual structural and cognitive social capital decreased the risk of being HIV infected. This study confirms previous qualitative studies indicating that access to structural and cognitive social capital is protective to HIV infection.

**Conclusions:**

We suggest that policy makers and programme managers of HIV interventions may consider strengthening and facilitating access to social capital as a way of promoting HIV preventive information and interventions in order to reduce new HIV infections in Tanzania.

## Background

Several studies have examined the association between poverty, gender inequality, sexual behaviors and HIV infection to explain the development of the HIV/AIDS epidemic [1–3]. Recently the framework of social capital has emerged to understand how social networks and norms may contribute to the spread of HIV. In this paper, we present results from a population-based study conducted in 2010 in Kagera region, Tanzania. Initially this region had a high level of HIV transmission as shown by the HIV prevalence and incidence in the late 1980s, which subsequently declined to low prevalence in the late nineties [4]. Inspired by these developments, the Kagera AIDS research project (KARP) designed a study to estimate the impact of social capital on the likelihood of being HIV infected.

Tanzania is among the few African countries that have registered a decline in HIV prevalence. In 2007–2008 the HIV prevalence in Tanzania among adults aged 15–49 years was estimated at 6.2% compared to 7.7% in 2003–2004 [5]. Several factors have been suggested to explain this decline. Firstly, the government showed its commitment to fight the epidemic by announcing HIV/AIDS as a national disaster indicating a political will as well as commitment to allocate more resources to prevent further spread of the epidemic. Secondly, the government implemented several interventions in fighting the epidemic including integrating HIV and AIDS in the long-term poverty reduction strategy. AIDS- and multi-sectoral committees were established in all sectors and administrative units to provide substantial support for communities in the fight against the HIV epidemic. Thirdly, the government created the portfolio of a Deputy Minister for Disasters and HIV and AIDS under the Prime Minister’s Office and allowed many civil society organizations to be registered to involve themselves in the fight against the epidemic. Lastly, the government increased the funding for HIV/AIDS activities both from government and donor resources and ensured that substantial funds reached the communities.

In monitoring the trends of the epidemic, the government through the National AIDS Council Programme (NACP) expanded a HIV sentinel surveillance system and conducted repeated surveys on HIV and sexual behaviour and included HIV and AIDS variables in the Demographic and Health Survey and the Tanzania HIV Indicator Survey. With regards to prevention, NACP initiated treatment of sexually transmitted infections in all hospitals and health centres and 60% of all dispensaries and ensured availability of voluntary, counseling and testing (VCT) and Prevention of mother to child transmission (PMTCT) services as well as availability of condoms [6].

Non-governmental organizations as well as the private and informal sectors became more active in providing HIV prevention and care services to their members and to the communities at large. The type of interventions, the target population, methods and coverage varied from one organization to another [7]. However, the HIV/AIDS prevention programmes focused their efforts on condom use, limiting the number of sexual partners/staying faithful to one partner, and delaying sexual debut in young persons. According to the 2007–2008 Tanzania HIV-Malaria survey [5], approximately 80% of the women and 90% of the men indicated that the risk of being HIV infected can be reduced by sticking to one uninfected partner. Furthermore, abstaining from sexual intercourse was the most frequently recognized prevention method among women and men aged 15–49 years (85% and 89% respectively).

In 1987, the Kagera AIDS Research Project (KARP) was initiated to study the epidemiology of HIV infection and to suggest appropriate interventions to fight the epidemic [8]. The initial study conducted in 1987 showed that four years after the discovery of the first HIV/AIDS cases, the urban areas of Kagera region had a HIV prevalence of 24% compared to 6.8% in the peri-urban area and 4.5% in the remote rural area. Based on the prevalence levels in 1987, Kagera was split into three geographical zones of high, medium and low HIV prevalence zones and followed using a series of cross-sectional studies to monitor the trends. These studies have indicated a downward trend of HIV infection in all zones; from 24% in 1987 to 18% in 1993, 13% in 1996, and 8.2% in 2004 in the high prevalence zone; from 10% in 1987 to 6.8% in 1996, and 4.3% in 1999 in the medium prevalence zone; and from 4.5% in 1987 to 2.6% in 1999 in a low prevalence HIV zone. The decline was largest among 15–24 year old women [4, 7, 8].

Studies in the region suggest that it is the synergistic effect of many different interventions that contributed to the decline in HIV prevalence [9–13]. These include distribution and use of condoms, information education and communication (IEC) strategies and spiritual and moral interventions by faith based organizations. Others include provision of counseling and testing services to the general population, adolescent sexual health interventions, interventions among groups most at risk, and provision of home based care services to people living with AIDS. Changes in sexual behavior involving increased condom use [7] as well as changes in attitudes and reduction of stigma associated with HIV infection coupled with the fear created by dying relatives [9] may have led to adoption of preventive sexual practices and hence decline in HIV prevalence.

Social capital can be defined as *“social networks, the reciprocities that arise from them, and the value of these for achieving (mutual) goals”* [14]. The concept has emerged as a conceptual framework to understand the ways through which people’s involvement in social networks may influence their health, including sexual related behaviors [15]. Social capital is basically operationalized by three interrelated elements of participation, trust and shared values [16, 17]. This implies that when people participate in social networks, they will develop common norms and values, which in turn enhance trust among members of the networks and later on such trust may extend to other people in the society [18].

Within the so called “social cohesion approach”, social capital is viewed as a collective asset, characterizing and benefitting a whole community by levels of social participation, reciprocity norms and trust [19, 20]. In this study, we utilized the so called “social network approach” to social capital, viewing it as an individual asset and described as *“the ability of actors to secure benefits by virtue of membership in social networks and other social structures”* ([21] p. 6). This implies that individual get access to certain benefits because of the relationships that they have built with others through participation in certain social groups and networks. Social capital can be further divided into structural and cognitive forms. According to Uphoff [22]*structural* social capital is about the forms and ways that social organizations and networks cooperate and interact while cognitive social capital includes norms, values, attitudes and beliefs that arise out of community interactions through social organizations and networks.

### Social capital and health outcomes

Several studies have demonstrated a direct relationship between access to both cognitive social capital (trust) and structural social capital (civic participation) and positive individual health, including psychological well-being, functional health, longevity, and good self-rated health [23–25]. Individual social capital is believed to promote health by ensuring access to social support and material resources as well as to role models for behavioural change. Further, social network involvement gives opportunities to learn new skills and develop a sense of belonging, which might have positive effects for health and health related behaviours [26, 27].

The association between social capital and sexual health behavior has also been well documented at individual level. In South Africa, participation in social clubs and community groups was associated with less risky sexual behavior among adolescents [28]. The pathway has been discussed by other scholars [29] who claimed that social capital may enhance empowerment among youths making them feel valued and respected, which in turn give them an opportunity to participate in making decisions pertaining to their lives including health issues. A recent cross-sectional study conducted to explore the association between social capital and abstinence, becoming faithful and condom use (ABCs) behaviors among Ugandan university students concluded that social capital was significantly associated with having less risky sexual behaviors [18].

In a previous qualitative paper [30], we explored different mechanisms through which social capital in the Kagera Region may have influenced sexual related behaviors. We hypothesized that social capital had facilitated the development of a conducive environment for members of social networks to reduce number of sexual partners, to abstain from sexual relations until marriage for the young generation, to have fewer opportunities for casual sex, and empowerment of community members to demand use of condoms. In another quantitative paper Gasto Frumence, Maria Emmelin, Japhet Killewo, Gideon Kwesigabo, Malin Eriksson, Lennarth Nystrom, ‘Social capital and HIV risk related behavior in Kagera region, Tanzania’ (unpublished) showed that high level of individual structural social capital increased the likelihood for a less risky sexual behaviour including use of condoms with casual partners. However, little is known about the influence of individual structural and cognitive social capital on the HIV prevalence. In this study we use data from a cross-sectional survey from Bukoba urban district to determine the HIV prevalence among sexually active adults aged 15–64 years and to examine the association between individual structural and cognitive social capital and socio-economic characteristics and the likelihood of being HIV infected.

## Methods

### Study area and sampling procedure

The study was performed in the Bukoba urban district, which in 1987 was defined as high HIV prevalence zone in Kagera region. Administratively, Bukoba urban district has only one division which is divided into 14 wards, each of which is further subdivided into several ‘streets’ that form the smallest administrative units. According to the 2002 national population census, the district had a total population of 81,221 with 19,259 households and the size of the household was estimated at 4.7. A four stage-cluster sample was drawn. In the first stage all 14 wards in the district were selected. In the second stage two streets in each ward were randomly selected making a total of 28 streets. In the third stage a total of 234 ten-cells clusters were selected with probability proportional to the size of the street. In the fourth stage one 15–64 year old adult was randomly selected within each household.

Since the main outcome measure in the survey was the prevalence of HIV infection, the sample size for the study was calculated based on the HIV prevalence estimate in the 2004 survey (8.4%), a desired precision level of 15%, a 5% significance level and a cluster design effect of 1.5 the final minimum sample size was 2787. Assuming a non-response rate of 10% the sample size for this study was estimated at 3066 households.

Information on population size and number of streets for each ward was received from the ward executive officer. Having established the total number of households in each ward and street, the total number of households in the ward were divided by the calculated minimum sample size of 3066 and obtained the sampling interval of 6. Then we divided the total number of households for each street by the sampling interval to obtain the number of households required from each street in the ward. After establishing the number of households to be selected from a street, the local leader was asked to provide the number of ten-cells together with their respective number of households in their jurisdiction. Using a ballot procedure the local leaders assisted and randomly selected ten cells until the required number of households per street was reached.

### Data collection procedure

The field team consisted of researchers and research assistants and their supervisors. Each selected individual was asked to give informed consent for an interview and a blood test following pre-test counseling. For the interview, a questionnaire containing information on socio- economic characteristics and structural and cognitive forms of social capital was administered by the first author and field assistants. The questionnaire was translated into Swahili to facilitate communication during interviews between respondents and field assistants. For the blood test, a blood sample was collected from each consenting individual and was later tested for HIV-1 antibodies. Data collection and blood test for this study were done between September 2010 and January 2011.

### The questionnaire

The questionnaire was designed to capture information on both structural and cognitive social capital indicators: voluntary participation in public works and participation in formal and informal organizations, reciprocity, and trust. We also included questions on socio-economic indicators as well as those related to HIV testing.

### Measurement of individual social capital

For measuring individual social capital we also applied factor analysis (using principal components analysis) with varimax rotation in order to determine the underlying items and their related dimensions. The first two components that accounted for 42% of all observed variance were retained because they obtained an eigenvalue >1. Table 1 shows that in the first component: individual ability to influence decisions, receiving financial support when in need, membership in organizations, giving support to others and interactions with neighbors, which reflect the structural form of social capital, had high loadings. Factors in this component were categorized as *structural* indicators of individual social capital indicating that networking form an important measure of individual structural social capital. In the second component, having general trust and trusting strangers had high loadings which reflected the *cognitive* form of social capital supporting the argument that these conventional dimensions are also important in measuring individual social capital.

**Table 1:** **Summary of indicators used to measure individual social capital**

Social capital indicators		Factor loadings components	
		1	2
*Networks and reciprocity (structural)*	Individual influence on decisions	0.63	
	Receiving financial support	0.59	
	Membership in organizations	0.57	
	Giving social support	0.55	
	Interaction with neighbors	0.45	
*Trust (cognitive)*	Trust in strangers		0.74
	General trust		0.73

### Laboratory procedures and evaluation of the testing strategy

Blood samples were collected aseptically in 5 ml red top vacutainers (BD, NJ, USA) and left to clot. Sera specimens were separated after centrifugation, aliquoted into 2 ml cryotubes tubes (Nalge Nunc International, IL, and USA) and stored at −20°C until the time for assay.

HIV status was determined by ELISA tests. Abbott Murex Wellcozyme anti-HIV-1 recombinant was used as first ELISA. Specimens with negative results underwent no further testing and were considered negative. Reactive samples were retested by second ELISA test, Dade Behring Enzygnost anti-HIV-1/2. This assay detect both HIV-1/2 infections. All samples that were reactive on First and second ELISA were regarded as positive for IgG anti HIV antibodies. Inno-Lia HIV I/II immunoblot assay (Immunogenetics) was used as a reference method. Discrepant results between the two ELISAs were confirmed by western blot using Inno-Lia HIV I/II assay. The sensitivity of both ELISA methods used is 100%. Specificity for Abbott Murex Wellcozyme anti-HIV-1 and Dade Behring Enzygnost anti-HIV-1/2 was 99.4% and 100% respectively [31].

### Ethical consideration

Ethical approval was received from the research and publications committee of the Muhimbili University of Health and Allied Sciences. Permission to carry out the study was sought and given from the Kagera regional and Bukoba urban district authorities. At the community level, ward leaders were informed about the study aims, the data collection procedures and their permission was sought and given. Furthermore, informed consent was sought from the study participants after explaining to them the study purpose, methods and data collection procedures. The field assistants conducted pre- and post-test counseling during collection of blood samples and returning of HIV results respectively.

### Data management and analysis

The questionnaires were checked for completeness on daily basis. After coding data were computerised, checked and cleaned using Epi Info and data were analyzed using SPSS. Bivariate logistic regression analysis was performed to estimate the impact of social capital and socio-economic characteristics on the likelihood of being HIV infected calculating odds ratios (OR) and 95% confidence intervals (CI). Independent variables significant in the bivariate analysis were entered into the multiple logistic regression analysis to control for confounding factors. When exploring the effect of social capital on HIV/AIDS, we limited our analysis to include levels of structural and cognitive social capital and how they are associated with HIV infection as an outcome measure.

## Results

### Descriptive statistics

Out of 3586 respondents who were interviewed, 3422 (95%) responded fully and agreed to test for HIV infections. There were more women respondents (58%) compared to men (42%) due to more women only households than men only households. Out of 3422 respondents only 149 (4.4%) had no formal education and out of the 757 respondents categorized as others in the occupation, 244 (32%) were students.

The overall HIV prevalence was 9.8% (CI: 8.8-10.8) with women (11.6%, CI: 10.2-13.0) having a higher prevalence than men (7.4%, CI: 6.0-8.8). The HIV prevalence decreased with increasing access to high levels of individual structural social capital: 15.5% (CI: 13.5-17.5) for individual with access to low level of structural social capital, 11.4% (CI: 8.9-13.9) for those with medium level of structural social capital and 4.5% (CI: 3.2-5.8) for individual with access to high structural social capital (Table 2). Different pattern was observed for cognitive individual social capital whereas individual with access to low level of cognitive social capital were found to have higher HIV prevalence (17.9%, CI: 15.8-20.2), followed by individual with access to high level of cognitive social capital 7.0% (CI: 4.9-9.1) and medium level of cognitive social capital 3.6% (CI: 2.4-4.8). When stratified by sex, women with low access to individual structural and cognitive social capital had a higher prevalence of HIV infection (19.0%, CI: 16.1-21.9 and 20.7%, CI: 18.1-23.8) compared to women with high access (5.4%, CI: 3.3-6.7) and 8.4%, CI: 5.0-10.9). A similar pattern could be observed among men, i.e. those who had low access to structural and cognitive social capital had a higher prevalence (11.4%, CI: 8.5-13.5) and 13.2%, CI: 9.8-16.2) compared to those with access to high levels (2.8%, CI: 1.1-4.9 and 5.1%, CI: 2.3-7.7).

**Table 2:** **Number tested, number of HIV positive and prevalence of HIV infection by socio-economic characteristics, levels of individual structural social capital (ISSC) and individual cognitive social capital (ICSC) and sex in Bukoba Urban district, Tanzania September 2010 – January 2011**

	Females			Males			Total		
	Number tested	Number HIV positive	Prevalence (%)	Number tested	Number HIV positive	Prevalence (%)	Number tested	Number HIV positive	Prevalence (%)
*Age*									
15-24	670	45	6.7	502	6	1.2	1121	51	4.4
25-34	736	85	11.5	473	45	9.5	1079	130	10.8
35-44	371	72	19.4	276	30	10.9	545	102	15.8
45-64	220	29	13.2	174	24	13.8	341	53	13.5
*Marital status:*									
Never married	302	15	5.0	653	20	3.1	955	35	3.7
Ever married	1695	216	12.7	772	85	11.0	2467	301	12.2
*Education:*									
No education	83	20	24.1	66	5	7.6	149	25	16.8
Primary	1649	190	11.5	1034	87	8.4	2683	277	10.3
Secondary & above	265	21	7.9	325	13	4.0	590	34	5.8
*Occupation:*									
Professional	199	13	6.5	415	20	4.8	614	33	5.4
Business	515	75	14.6	305	18	5.9	820	93	11.3
Farmers	472	71	15.0	207	28	13.5	679	99	14.6
Housewife/unemployed	531	44	8.3	21	-	-	552	44	8.0
Others	280	28	10.0	477	39	8.2	757	67	8.9
*Religion:*									
Christian	1548	185	12.0	1126	91	8.1	2674	276	10.3
Moslem	449	46	10.2	299	14	4.7	748	60	8.0
*Wealth index:*									
Very poor	387	56	14.5	301	31	10.3	688	87	12.6
Poor	381	52	13.6	300	23	7.7	681	75	11.0
Moderate	408	62	15.2	276	22	8.0	684	84	12.3
Rich	388	32	8.2	297	20	6.7	685	52	7.6
Very rich	433	29	6.7	251	9	3.6	684	38	5.6
*ISSC*									
Low	690	131	19.0	587	67	11.4	1277	198	15.5
Medium	438	51	11.6	203	22	10.8	641	73	11.4
High	648	35	5.4	322	9	2.8	970	44	4.5
*ICSC*									
Low	760	157	20.7	433	57	13.2	1193	214	17.9
Medium	535	21	3.9	371	12	3.2	906	33	3.6
High	320	27	8.4	254	13	5.1	574	40	7.0
Total	1997	231	11.6	1425	105	7.4	3422	336	9.8

### Association between structural and cognitive social capital and HIV infection

Table 3 presents the results of the bivariate and multiple logistic regression analysis of the association between structural and cognitive forms of individual social capital and the likelihood of being HIV infected among sexually active 15–64 year old adults. Individuals with low and medium levels of structural social capital were almost four and three times likely to be HIV infected compared to those who had high levels of individual structural social capital (OR =3.9, CI: 2.8-5.4 and 2.7 CI:1.8-4.0). After adjusting for sex, age, marital status, level of education, occupation and wealth index the association between medium and high level of structural social capital and HIV positive became stronger (OR =8.6, CI: 5.7-13 and 2.9, CI: 1.9-4.7). This means that sex, age, marital status, level of education, occupation and wealth of individuals are important factors in determining the level of structural social capital. When stratified by sex, the results indicated that men who had low access to structural social capital had a higher risk of being HIV infected than those with high access (OR =4.5, CI: 2.2-9.1). This association was strengthened after adjusting for sex, age, marital status, level of education, occupation and wealth index (OR =9.1, CI: 4.3-19). A similar pattern was observed for women’s access to individual structural social capital and HIV infection (Table 4).

**Table 3:** **Bivariate and multiple logistic regression analysis of the association of socio-economic characteristics, access to individual structural social capital (ISSC) and individual cognitive social capital (ICSC) on the HIV prevalence in Bukoba Urban district, Tanzania September 2010 – January 2011**

Characteristics	Categories	Number of		Bivariate analysis		*Multiple logistic regression	
		Cases	Referents	OR	95% CI	OR	95% CI
Sex	Males	82	976	1		1	
	Females	205	1410	1.6	1.3-2.1	1.6	1.1-2.3
Age	15-24	51	1121	1		1	
	25-34	130	1079	2.7	1.9-3.7	2.3	1.5-3.7
	35-44	102	545	4.1	2.9–5.8	3.7	2.2-6.1
	45-64	53	341	3.4	2.3–5.1	2.8	1.6-4.9
Marital status	Never married	35	920	1		1	
	Ever married	301	2166	3.7	2.6-5.2	3.1	1.8-5.4
Education	Secondary and above	34	556	1		1	
	Primary	277	2406	1.9	1.3-2.7	0.98	061-1.6
	No education	25	124	3.3	1.9-5.7	1.4	0.71-2.8
Occupation	Professional	33	581	1		1	
	Business	93	727	2.3	1.5–3.4	1.4	0.81-2.3
	Farmers	99	580	3.0	2.0-4.5	1.4	0.80-2.4
	Housewife/unemployed	44	508	1.5	0.96–2.4	0.82	0.44-1.5
	Others	67	690	1.7	1.1–2.6	1.6	0.92-2.8
Religion	Muslim plus others	60	688	1			
	Christian	276	2398	1.3	0.98–1.8		
Wealth	Very rich	38	646	1		1	
	Rich	52	633	1.4	0.9–2.2	2.0	1.2-3.3
	Moderate	84	600	2.4	1.6–3.5	2.1	1.3-3.5
	Poor	75	606	2.1	1.4–3.2	1.9	1.2-3.2
	Very poor	87	601	2.5	1.7–3.6	1.3	0.79-2.3
ISSC	High	44	926	1		1	
	Medium	73	568	2.7	1.8-4.0	2.9	1.9-4.7
	Low	198	1079	3.9	2.8-5.4	8.6	5.7-13.0
ICSC	High	40	534	1		1	
	Medium	33	873	0.51	0.31-0.81	0.37	0.22-0.62
	Low	214	979	2.9	2.0-4.2	2.4	1.6-3.5

Odds ratio (OR) and 95% confidence interval (CI).

*Adjusted for sex, age, marital status, level of education, occupation and wealth index.

**Table 4:** **Logistic regression and multiple logistic regression analysis of the association of access to individual structural social capital (ISSC) and individual cognitive social capital (ICSC) and the likelihood of being HIV-infected stratified by sex in Bukoba Urban district, Tanzania September 2010 – January 2011**

Characteristics	Categories	Number of		Bivariate analysis		*Multiple logistic regression	
		Cases	Referents	OR	95% CI	OR	95% CI
Females ISSC	High	35	613	1		1	
	Medium	51	387	2.3	1.5-3.6	2.4	1.5-3.7
	Low	131	559	4.1	2.8-6.1	7.1	4.7-10.9
Males ISSC	High	9	313	1		1	
	Medium	22	181	4.2	1.9-9.4	5.1	2.3-11.5
	Low	67	520	4.5	2.2-9.1	9.1	4.3-19.1
Females ICSC	High	27	293	1		1	
	Medium	21	514	0.44	0.25-0.79	0.44	0.24-0.81
	Low	157	603	2.8	1.8-4.4	2.7	1.7-4.2
Males ICSC	High	13	241	1		1	
	Medium	12	359	0.62	0.28-1.4	0.75	0.33-1.7
	Low	57	376	2.8	1.5-5.2	3.2	1.7-6.1

Odds ratio (OR) and 95% confidence interval (CI).

*Adjusted for sex, age, marital status, level of education, occupation and wealth index.

As seen from Table 3 the study findings indicate a weaker association between high level of cognitive social capital and HIV infection than for structural social capital. The association weakened after controlling for socio-economic confounding factors but remained statistically significant (OR =2.4; CI: 1.6-3.5). Having access to medium level of cognitive social capital did not have any effect on the likelihood of being HIV infected. When stratified by sex, the results indicated a similar pattern for men and women (Table 4). However, when adjusting for socio-economic variables, the risk for being HIV infected given access to low cognitive social capital increased for men but decreased for women, still statistically significant (OR =3.2; CI: 1.7-6.1 and OR =2.7; CI: 1.7-4.2).

## Discussion

Bukoba urban district is characterized by high HIV prevalence compared to other districts in Kagera region. The study findings have shown unequal distribution of structural and cognitive social capital between women and men. 37% and 29% of women and men respectively had access to high level of individual structural social capital while 24% among men 20% among women had access to high level of cognitive social capital. Our previous qualitative study in the same study setting [32] also showed that women were more active in participating in formal and informal organizations compared to men. Similar findings were observed in the United Kingdom where there has been sustainability in social capital because of increasing number of women’s participation in various associations compared to men [33]. In this study we found that more men than women had high access to cognitive social capital *(measured by general trust and trust in strangers)*. This can partially be explained by the fact that traditionally men in African context have been more exposed to the formal and informal employment opportunities, business ventures and recreational activities which may enhance their ability to trust people with whom they regularly get in contact with.

### The association between structural form of social capital and HIV infection

This study suggests that in an urban Tanzania’s context, access to high level of individual structural and cognitive social capital decreased the risk for HIV infection. Women and men who have low levels of structural individual social capital were more likely to be HIV positive compared to those who have high structural individual social capital, and this association was stronger for men. This means that participation in informal and formal organizations, giving social support to others who are in need, interact with neighbors by visiting them when they get sick and the ability to influence decisions affecting someone’s daily life (all forming structural individual social capital) may have played a positive role in influencing sexual behaviors for individuals with high levels of structural individual social capital. As explained in our previous qualitative paper [30] social capital in the structural form have been utilized as active avenues for community members to come together and empower each other thereby creating an enabling environment in which members could adopt safe behaviors to protect themselves from HIV infection. These safe behaviors included among others reduction of numbers of sexual partners, abstinence from engaging in sexual relations until marriage, use of condoms and caused fewer opportunities for casual sex. Our findings that structural individual social capital has a protective effect on HIV infection is consistent with findings from an exploratory study investigating links between sexual health and social capital in a South African mining community [29]. They reported that young women and men, particularly those participating in youth organizations, were less likely to be HIV positive due to the fact that these organizations focused on activities that empower youth in terms of social skills, personal ambitions and confidence. The Uganda’s success in the drastic declining trends of HIV infection has been closely associated with effective social mobilizations including networks involving peer groups [34]. Campbell [29] also showed that men who belong to faith based organizations particularly churches increased their likelihood of having no casual sex partners, a protective behavior against HIV infection. The Christian teachings particularly those norms emphasizing on practicing monogamism has also been associated with men’s reduction of number of sexual partners and sticking to only one partner. In the United States, young people who were actively participating in social group activities such as those organized within the church, schools or community where they belong were found to have reduced HIV risk sexual behaviors [35] while others were less likely to have engaged in sexual intercourse [36].

However, high level of structural social capital has also been associated with increasing the risk of being HIV infected. High levels of interactions have been reported to be a source of practicing HIV risk behaviors. According to Campbell [37], young men and women who were members of voluntary saving clubs commonly known as stokvels*,* were more likely to drink alcohol, which is regarded as a risk factor for risky sexual behaviour and thereby HIV infection. Similarly young men belonging to these groups were more likely to be infected with HIV, while both adult and young women belonging to stokvels were more likely to have had a casual partner. Other networks such as injecting drug users (IDUs) and commercial sex workers (CSW) have been reported to be a conduit of HIV infection. The IDUs are claimed to be risk networks because of syringe sharing among the networks members [38] while in India CSW have been reported to form a core risk network for HIV infection since they are more likely to engage in unprotected sex with their clients because of poverty resulting into inability to negotiate about condoms [39].

### The association between cognitive form of social capital and HIV infection

Our findings show high level of cognitive individual social capital as measured by general trust and trust in strangers was associated with low risk for being HIV infected. Men and women with low cognitive social capital were more likely to be infected with HIV compared to men and women with high levels of cognitive social capital. Nevertheless, this implies that individuals who have high level of cognitive social capital are more likely to trust and practice protective health behaviors in the form of norms, values and rules of conducts either developed or practiced within the social networks or the community where they belong. Such protective health related behaviors may include those which have been reported in our previous studies including religious teachings about abstinence and faithfulness. We also showed that high level of trust in religious leaders in the community is associated with accepting their teachings and resulting in practicing protective behaviors to HIV infection. Other norms and values were developed by social groups emphasizing reductions of alcohol intake among group members while the community members, particularly parents, were concerned about abolition of HIV risk environment such as night parties including discos for youth. Furthermore, when groups members develop their own rules of conduct and stipulate punishment for failure to abide with such rules, this may have motivated members to behave in a way that is HIV protective like reducing number of sexual partners [30].

Findings from other studies [40, 41] reported that norms and values guiding and circulating within social capital have a positive influence on members’ health. In South African contexts, community groups and social clubs have been commended for playing a protective role against HIV infection by creating social norms such as accepting use of condoms [28]. Our findings that high level of cognitive social capital decreased the likelihood of being HIV infected is consistent with findings from another South African study which underscored that living in households with high levels of cognitive social capital (characterized by a sense of collective mobilization concerning common issues, mutual support, and reciprocity) was linked to higher use of condoms and lower HIV prevalence among both men and women [42].

### Health policy implications

Policy provides directions and guidance on how both human and non-human resources can be re-distributed within the society for effective implementation of the various programmes including HIV prevention. It is believed that a good policy must be evidence based; implying that it should obtain information from empirical based findings [43]. For the past years, our health policy makers have been entirely relying on information related to conventional ways of preventing diseases. The main focus has been on developing strategies on how to prevent individual risks that expose people to diseases including those related to HIV epidemic and maintaining the good behaviors for healthy population. This conventional approach is being criticized for ignoring the social context, which either act as a buffer or a facilitator for a healthy population [44]. In Tanzania, it is estimated that there are about 2 million adults aged 15 years and above who were living with HIV/AIDS [6]. In light of these numbers, health policy must address the social context in which HIV risk behaviors occur including social capital. This work may assist policy makers in the design and delivery of HIV prevention intervention programs by taking into consideration strategies to mobilize structural social capital in the form of informal and formal social organizations and networks. Empowering formal and informal organizations and networks may entail as Pronyk and his colleagues [42] suggested, a provision of training and mobilization programmes to these organizations.

In Tanzania’s context, the mobilization programmes should involve local government and non-governmental organizations leaders who may motivate the non-members of the existing social groups and networks to join into these organizations. These leaders may utilize the existing social capital as avenue for conveying important messages and information regarding HIV prevention instead of relying only on conventional methods such as distribution of fliers, posters, use of television and radio or news papers and pamphlets. In the African context, not everyone is accessible to these modern types of communication channels and many people even cannot read and write, hence only the literate and wealthy part of the community are accessing these kinds of preventive messages or information. Such limitations of the conventional approach to HIV prevention support the need for a ‘paradigm shift’, which Beeker and his colleagues [44] suggest for understanding the social determinants of HIV transmission and increasing community capacity in the development and implementation of sustainable HIV prevention programmes. Facilitating the involvement of social groups or certain community members to participate in the design and delivery of a particular HIV prevention intervention is one strategy to acknowledge the broader social determinants in HIV intervention. Such programs are more likely to be successful since it increases people's control over their daily life [45].

This study support the argument raised by the Grootaert and Bastelaer [46] that when social capital is used as channel of diffusing information to the community, it increases possibility of cooperation and sharing, as well as lowering the cost involved in information, education, and communication. Given the fact that in Tanzania and many other African countries, dissemination of information, education and communication about HIV prevention, has been involving massive expenditure of resources including money, making it difficult to reach all vulnerable population especially in the rural areas, the suggested paradigm shift of using social capital in the design and delivery of HIV interventions could be less costly and therefore a feasible and reliable channel of communication.

### Study strengths and limitations

This study utilized a cross-sectional study design and the sample size was large enough to calculate statistical power as well as for assessing the effect modification without risking a type two error. We believe that the study does not have selection bias given the high response rate (95%). We controlled our analyses for potential confounding factors such as age, sex, level of education, occupation, marital status and wealth index since other studies have reported that social capital may be associated with socio-economic factors. The strengthening of the association between individual structural social capital and HIV infection in the multivariate analysis after controlling for socio-economic confounding factors is an indication that there is a strong independent association between individual structural social capital and HIV infection. Some cases might over report access to individual social capital especially regarding participation in social groups as community members would expect that being an active member in a group would mean accessing formal loan from the government or other organizations. This is due to the fact that in Tanzania, both the central and local governments encourage people to form social groups as a means for them to access loans. Over reporting of access to individual structural social capital may cause bias to our finding. To our knowledge, asking about general trust and trust in strangers in African context where urban based societies are faced with high level of criminals, theft, hooliganism and dishonest people, may affect the way people have responded to this question and ultimately the level of the trust. However, research assistants were well trained on how they should approach respondents regarding issues related to trust in order to remove ambiguity.

## Conclusions

The results presented here from a district which witnessed declining trends in HIV infection in Kagera Tanzania, add important evidence supporting the argument that social capital (measured in terms of structural and cognitive forms) may have played a protective role in this trend. The study found that high level of individual structural and cognitive social capital was associated with low HIV prevalence as compared to low level. This means that individuals who had low level of structural and cognitive social capital were vulnerable to HIV infection. Hence, policy makers and implementers of HIV prevention interventions need to consider inclusion of social capital in the design and delivery of effective HIV and AIDS interventions strategies.
